# Effectiveness of person-centered intervention on obstetric violence during facility-based childbirth among women who delivered in public hospitals in Southwest Ethiopia

**DOI:** 10.3389/fpubh.2025.1510430

**Published:** 2025-07-17

**Authors:** Ayanos Taye, Tefera Belachew

**Affiliations:** ^1^School of Nursing, Faculty of Health Science, Institute of Health, Jimma University, Jimma, Ethiopia; ^2^Department of Nutrition and Dietetics, Faculty of Public Health, Institute of Health, Jimma University, Jimma, Ethiopia

**Keywords:** obstetric violence, person-centered intervention, childbirth, hospitals, Ethiopia

## Abstract

**Background:**

Obstetric violence (OV) is a significant public health issue affecting reproductive health services and maternal health outcomes. Despite studies documenting its prevalence in Ethiopia, no experimental studies have assessed intervention effects. This study evaluates person-centered interventions for OV in public hospitals in Southwest Ethiopia, using an experimental design.

**Methods:**

A quasi-experimental study was conducted in Southwest Ethiopia involving 396 women, divided into a control group (CG; *n* = 198) and an intervention group (IG; *n* = 198). The intervention group received person-centered interventions, including respectful maternity care workshops and maternal recognition certificates, while the control group received standard care. Statistical analyses included *t*-tests and regression to assess the intervention’s impact on OV.

**Results:**

The proportion of women who utilized companions during facility-based childbirth was higher in the intervention group [25.8%; 95% confidence interval (CI): 19.6, 31.9%] than in the control group (12.1%; 95% CI: 7.5, 16.7%). The proportion of women who experienced OV decreased significantly in the intervention group compared with the control group (IG: 42.05 ± 6.97; CG: 66.45 ± 12.12; *p* < 0.000), with a 26.00-point decrease in the experimental group. The multivariable general linear model revealed that the mean difference (MD) of non-confidential care (MD = −3.28; 95% CI: −3.66, −2.90), undignified care (MD = −7.03; 95% CI: −7.76, −6.31), non-consented care (MD = −5.64; 95% CI: −6.35, −4.92), physical abuse (MD = −4.80; 95% CI: −5.30, −4.31), discrimination (MD = −3.37; 95% CI: −3.79, −2.94), and detention (MD = −0.28; 95% CI: −0.51, −0.05) were significantly reduced in the intervention group, with effect sizes of 0.421, 0.480, 0.380, 0.479, 0.382, and 0.014, respectively. Women in the intervention group had an OV score that was, on average, 23 points lower (*β* = −23.42; 95% CI: −25.40, −21.44) than those in the control group. Additionally, women in the intervention group who had frequent contact with healthcare providers experienced, on average, a seven-point lower OV score (*β* = −7.47; 95% CI: −4.055, 18.37) than those in the control group.

**Conclusion:**

This study revealed that implementing person-centered interventions through respectful maternity care workshops, maternity open days, and maternal certificates of recognition significantly decreases the incidence of OV and ultimately promotes respectful maternal care, improving maternal healthcare services.

## Introduction

Obstetric violence (OV) is a form of disrespect and abuse toward women during childbirth, which involves non-confidential care, undignified care, physical and verbal abuse, emotional mistreatment, as well as neglect and violations of privacy and autonomy within healthcare facilities (1). OV is an increasingly recognized global health concern that violates the universal right of childbearing women (1–3). Recent data indicate that global OV varies by country, with the lowest rates in developed countries and the highest rates in developing countries (1, 2, 4). In developed countries, studies have shown a prevalence of 15% in the USA and 78.4% in Italy, while in developing countries, the prevalence is significantly higher, ranging from 74 to 98.9% in Ethiopia and 98% in Nigeria (1, 2, 4, 5). The highest prevalence of OV was documented during facility-based childbirth rather than during other maternal healthcare services, such as antenatal care (ANC) and postnatal care (4, 6). OV remains an important public health problem among facility-based childbirth mothers, negatively impacting their health-seeking behavior and trust in healthcare systems. This leads to adverse maternal and child health outcomes, such as obstetric complications and severe psychological issues (2, 7–9).

Numerous studies have highlighted that the most common factors that cause OV are multifaceted and originate from issues within the healthcare system, women’s knowledge of their reproductive rights and attitudes toward healthcare, as well as insufficient training and accountability among healthcare professionals (4, 10, 11).

Although the magnitude of the OV has increased alarmingly, and predictors have been identified in African regions, particularly in Ethiopia, less attention has been given to mitigating OV during institutional delivery over recent times (2, 4).

OV mitigation strategies have received global attention because of their significant impacts on women’s health and rights (2, 12–18), and few OV interventions, such as training healthcare providers, improving facility conditions, community engagement, and patient empowerment, have been implemented in the last decade, with some progress. However, the percentage of women experiencing OV has remained unchanged over recent years, and its prevalence is also well documented (2). There is a need to implement interventions to reduce the pervasive incidence of OV in Ethiopia, particularly in study settings, through provider training on RMC and women’s empowerment (19, 20).

Respectful maternity care is one of the strategies that effectively engages healthcare providers and women in OV reduction programs (19). In Ethiopia, although the Federal Ministry of Health launched the Compassionate, Respectful, and Caring (CRC) initiative, which focuses on healthcare providers, a significant improvement has not been observed. The few interventional studies conducted to lessen OV or disrespect and abuse have not been meaningfully limited because of the undermining of women’s participation in interventions or programs (18, 21).

Although person-centered interventions (respectful maternity care training, maternity open days and a maternal certificate of recognition) effectively contribute to the reduction of OV during facility-based childbirth through empowering women, enhancing provider attitudes, and promoting a culture of respect within maternity services, no evidence shows the combined effect of these interventions on OV in Ethiopia. Therefore, this study examined the impact of person-centered interventions on OV among mothers in public hospitals in Southwest Ethiopia.

## Methods

### Study design and settings

A quasi-experimental study was conducted among women who delivered in public hospitals (specifically, Seka Hospital and Agaro Hospital) in Southwest Ethiopia from 2021 to 2022. These two hospitals primarily focus on maternal and child health for large communities residing in rural areas and select urban communities.

### Participants and procedures

The study was conducted among 396 women, aged 19–49 years, who had delivered in hospitals. These women were selected based on specific inclusion and exclusion criteria. Women who delivered at the study sites and were between the ages of 19 and 49 were included in the study. Conversely, women who underwent a cesarean section and experienced complications were excluded from the study. The sample size was calculated using ClinCalc software based on the following assumptions: a prevalence of disrespect and abuse among women (P1) of 91.7% (22), a reduction in the proportion of disrespect and abuse among women (P2) of 10% in the intervention, a confidence level of 95%, a power of 80%, and a margin of error of 5%. A sample size of 360 (180 each in the intervention and control groups) was calculated. Considering the non-retrieval rate of 10% for each arm, the final sample size was 396 (198 each in the intervention and control groups). A simple random sampling technique was used to recruit the study participants who fully met the eligibility criteria. Next, a proportional allocation to the sample size was performed based on the average number of client flows that the two hospitals had registered in the last 6 months preceding the survey. The study was conducted in three phases: the preintervention phase (baseline data collection), the intervention phase (intervention implementation), and the postintervention phase (endline data collection).

### Preintervention phase (baseline data collection)

A pretested, validated, and structured interview questionnaire was used to collect actual data from the study participants over 2 months. The tools include various factors, such as sociodemographic characteristics, obstetric-related factors, and the practice of OV.

### Intervention phase (implementation)

A person-centered intervention, comprising a Respectful Maternity Care (RMC) workshop, Maternity Open Days (MOD), and a Maternal Certificate of Recognition (MCOR), was implemented at the intervention site. A RMC workshop was conducted for healthcare providers working in Maternal and Child Health (MCH) units. The workshop aimed to equip these professionals with the skills and knowledge necessary to deliver respectful and dignified intrapartum care. The workshop was provided based on the compassionate and respectful care manual developed by the Ethiopian Federal Ministry of Health and other manuals developed for this purpose (23–25). The guide contains different contents, such as an overview of maternal health, the nature of OV, human rights (specifically women’s rights) and childbearing rights, the promotion of respectful and dignified care during childbirth, professional ethics, value clarification, and attitudinal change and implementation of MODs. The draft manual was reviewed by three senior maternal health experts from Jimma University and the Jimma Zonal Health Department for its content and applicability to the local context. The workshop was delivered through various teaching methods, including interactive presentations, group discussions, role-playing, and brainstorming. The RMC workshop was held over 3 consecutive days with a total of 16 midwives (12 women and 4 men) participating in two rounds.

Maternity Open Days (MODs) served as another intervention strategy designed specifically for women. These events invited pregnant women to participate in various activities, including intrapartum education. This included orientations on OV, universal rights of childbearing women, and responsibilities during childbirth. Additionally, participants received an introduction to Basic Safe Childbirth Supplies Orientation (BSCSO), which involved demonstrating essential items required for safe and hygienic labor and delivery management, as outlined in the WHO Safe Childbirth Checklist. Facility Tours: Participants visited Maternal and Child Health (MCH) units, focusing on labor, delivery, and postnatal rooms. Trained providers conducted these interventions, inviting pregnant women to attend according to their scheduled antenatal care (ANC) follow-ups. Given that the median gestational age at the first ANC visit is 30 weeks, as reported in the EDHS 2016 report, the MOD events targeted third-trimester pregnant mothers with a gestational age of 30 weeks or those who had completed their third ANC visit. On average, approximately 25 pregnant women participated in these events every 3 weeks over 24 weeks. The teaching materials were developed and translated into the local language by experts. Supervisors and the research team documented the activities during the MODs.

The third component of the person-centered intervention is the Maternal Certificate of Recognition (MCOR), which was awarded to women. This recognition was conducted at each MOD event, which were held every 3 weeks over a period of 24 weeks, resulting in a total of 8 events. At each event, three women received the certificate, acknowledging that a total of 24 mothers were awarded this recognition, recognizing those who had adhered to the principles of the continuum of maternal healthcare. This intervention acknowledges and empowers women by validating their experiences with maternal healthcare services. It has the potential to enhance interactions with healthcare providers by fostering trust and contributing to a more respectful environment of maternity care. The recipient of this recognition is referred to as a “Hero Mom.”

### Postintervention phase (endline data collection)

The postintervention phase was conducted during the period following the implementation of the intervention in the study setting. This phase is vital for assessing the effects and outcomes of the intervention. Specifically, it evaluates whether the person-centered intervention has successfully reduced OV. Additionally, the levels of OV observed in the postintervention phase were compared with those from the baseline phase to determine if significant changes occurred. A structured questionnaire, administered by an interviewer during baseline data collection, was used to collect the endline data.

**Figure 1:**
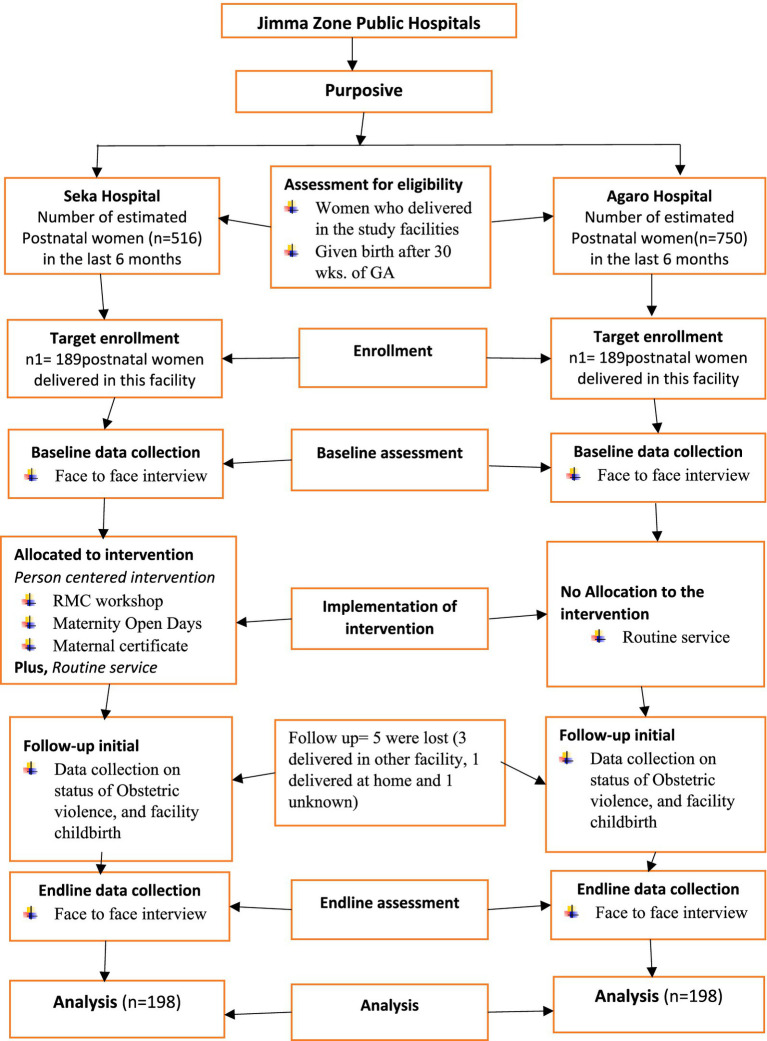
Consolidated standards of reporting trials (CONSORT) diagram of the study design.

This figure depicts the sampling method, allocation of study units, and assessment of eligibility criteria to include study participants (Figure 1).

### Data collection tools and methods

Data related to OV were collected via standardized survey questionnaires developed for similar purposes from different relevant studies, such as the Maternal and Child Survival Program (MCSP), Maternal and Newborn Programs, and research conducted in Kenya and Tanzania (13, 16). The questionnaires included sociodemographic and economic factors, obstetric-related factors, healthcare provider-related factors, and information related to OV (outcome variables). The experts first translated the questionnaires into the local languages (Afan Oromo and Amharic) and then back-translated them into English to ensure consistency in meaning and intentions. Data were collected by trained data collectors through face-to-face interviews. Enumerators and supervisors received 2 days of training on the purpose of the study, data quality assurance, ethical issues, identification of possible challenges, and effective communication strategies. The supervisors and research team closely monitored the data collection process and addressed any issues that arose to maintain data quality. Eligible postpartum women were interviewed in a private room to ensure their privacy and confidentiality.

### Measurements

OV is a violation of human rights, emphasizing disrespect, abuse, and neglect in maternity care settings (26). It was measured using the Bowers and Hill categories to assess disrespect and abuse, which were first developed by Sheferaw et al. (43) in Ethiopia, demonstrating its reliability (α = 0.845). This instrument consists of six domains and 29 items, including non-confidential care (4 items), non-consented care (4 items), undignified care (10 items), discriminatory care (4 items), detention in healthcare facilities (2 items), and physical abuse (5 items). A 5-point Likert scale ranging from “never: 1” to “always: 5” was used. The overall OV score was calculated by summing the responses to all items. Negatively worded statements were scored negatively. High composite scores on this scale indicate that participants experienced OV during childbirth (see Table 1).

**Table 1:** Domains of obstetric violence (OV) manifestations.

OV domains	Manifestations/specifications
Non-confidential care	Sharing private information with the women, exposing the mother’s body during childbirth, and a lack of visual barriers (such as cloths, blankets, or screens) during delivery.
Undignified care	Shouting or scolding, unfriendly welcoming, disrespectful treatment, negative or disparaging comments about women, Use of language that is not easily understood by women, and failure to inform women about alternative birth positions.
Non-consented care	Failing to provide information before and during procedures, neglecting to obtain the mother’s consent beforehand, and not encouraging women to ask questions about their care.
Physical abuse	Hitting, slapping, pushing, pinching, pushing, tied to the delivery bed, restraining, and failure to adequately manage the mother’s pain.
Discrimination	Mistreating women based on social class, poverty, ethnicity, religion, tribe, age, marital status, or health status. Leaving women alone during labor and prohibiting the presence of a companion during labor and childbirth.
Detention	Unnecessary detention of women in healthcare facilities due to inability to pay medical expenses and other reasons.

OV, obstetric violence.

Principal component analysis (PCA) is widely used to construct wealth indices by analyzing household asset data. Filmer and Pritchett stated that principal component analysis estimates wealth levels effectively and provides a comprehensive and concrete economic measure (27). Thus, a total of 18 survey items about household assets were utilized to determine the level of individuals’ income, ranging from the lowest to the highest.

### Data analysis

The data were entered into EpiData software and then analyzed using IBM Statistical Package for the Social Sciences (SPSS) version 26.0 software, which uses descriptive statistics such as “number, percentage, mean, standard deviation.” Chi-squared tests for categorical variables and independent sample *t*-tests for continuous variables were used to confirm the differences in demographic and obstetric characteristics between groups. Principal component analysis (PCA) was employed to compute household wealth. Independent sample *t*-tests were used for intergroup comparisons of OV and its typologies, and paired sample *t*-tests were used for intragroup comparisons. A bivariate and multivariable general linear model was used to determine the effect size of the intervention on typologies of OV. Bivariate general linear modeling was used to estimate the associations between predictors and OV via the beta coefficient. Statistical significance was considered at *p* < 0.05.

### Ethical consideration

Approval for the study was obtained from the Jimma University IRB ethics committees before the commencement of data collection. All procedures and methods were conducted in compliance with the relevant guidelines and regulations. The researcher was obligated to provide prospective participants with sufficient information to enable them to make informed decisions to participate in the study. Written informed consent was obtained after the participants agreed to participate, having been informed about the purpose, benefits, and risks of the study, as well as the process of data collection and confidentiality. For this study, participants were informed that their participation was voluntary and that they would not face any form of coercion or influence. The participants were informed that they could withdraw at any time without giving a reason and that there was no penalty for withdrawal.

## Results

As listed in Table 2, 92.42 and 93.94% of the 198 study participants in the intervention group and control group, respectively, were within the age range of 19–34 years. Over half (57.07%) of the participants were from rural areas in the intervention group and in the control group (63.64%). The majority of the study participants were Muslim by religion in both the intervention (80.81%) and control (71.72%) groups. More than 90% of the women in the intervention group (93.43%) and control group (92.42%) were married. Regarding the educational status of the women, a large proportion had completed their primary education or higher in both the intervention group (69.20%) and the control group (60.10%). The economic status of the women differed significantly between the two groups (*p* < 0.001), with a lower proportion of women with low income or experiencing poverty was lower in the intervention group (12.6%) than in the control group (28.8%).

**Table 2:** Sociodemographic/economic characteristics of postpartum women in the intervention and control groups in Southwest Ethiopia, 2024.

Variables	Categories	Intervention (*n* = 198)	Control (*n* = 198)	*p*
		*n* (%)	*n* (%)	
Age (years)	19–20	34 (17.17)	51 (25.76)	0.110
	21–34	149 (75.25)	135 (68.18)	
	35–49	15 (7.58)	12 (6.06)	
Residence	Rural	113 (57.07)	126 (63.64)	0.182
	Urban	85 (42.93)	72 (36.36)	
Religion	Muslim	160 (80.81)	142 (71.72)	0.194
	Orthodox	24 (12.12)	37 (18.69)	
	Protestant	13 (6.57)	17 (8.59)	
	Other*	1 (0.51)	2 (1.01)	
Marital status	Never married	7 (3.54)	7 (3.54)	0.862
	Married	185 (93.43)	183 (92.42)	
	Divorced	6 (3.03)	8 (4.04)	
Educational status	No education	61 (30.81)	79 (39.90)	0.144
	Primary	34 (17.17)	37 (18.69)	
	Secondary	78 (39.39)	66 (33.33)	
	More than secondary	25 (12.63)	16 (8.08)	
Occupational status	Housewife	143 (72.22)	126 (63.64)	0.423
	Farmer	17 (8.59)	19 (9.60)	
	Business	14 (7.07)	22 (11.11)	
	Student	9 (4.55)	11 (5.56)	
	Employed	15 (7.58)	20 (10.10)	
Wealth	Lowest	25 (12.6)	57 (28.8)	<0.001
	Second	26 (13.1)	50 (25.3)	
	Middle	35 (17.7)	44 (22.2)	
	Fourth	50 (25.3)	30 (15.2)	
	Richest	62 (31.3)	17 (8.6)	

*Catholic and pagan.

There was a significant difference in the number of antenatal care contacts between the intervention group and the control group (*p* = 0.019). The proportion of women who had four or more antenatal care contacts was greater in the intervention group [87 (43.9%)] than in the control group [60 (30.3%)]. Approximately three-fourths of the study participants in both groups did not experience any obstetric complications during facility-based childbirth. Regarding the intentions of women to have more children, the majority of respondents in both the intervention group (88.4%) and the comparison group (85.4%) wanted to have more children. Regarding companion utilization, there was a significant difference between the intervention group (25.8%) and the control group (12.1%) (*p* < 0.001). Conversely, the intervention group exhibited a significantly lower absence rate of companions (74.2%) compared to the control group (87.9%) (*p* < 0.001) (Table 3).

**Table 3:** Obstetric characteristics of postpartum women in the intervention and control groups in public hospitals in Southwest Ethiopia, 2024 (n1 = n2 = 198).

Variables	Categories	Intervention *n* = 198	Control *n* = 198	*p*
		*n* (%)	*n* (%)	
ANC contact	1	18 (9.1)	22 (11.1)	0.019
	2–3	93 (47.0)	116 (58.6)	
	≥4	87 (43.9)	60 (30.3)	
Birth order	1	67 (33.8)	57 (28.8)	0.014
	2–3	76 (38.4)	89 (44.9)	
	4–5	31 (15.7)	43 (21.7)	
	≥6	24 (12.1)	9 (4.5)	
Time of birth	Day time	62 (31.1)	72 (36.4)	0.288
	Nighttime	136 (68.7)	126 (63.6)	
Obstetric complications during delivery	Yes	39 (19.7)	45 (22.7)	0.461
	No	159 (80.3)	153 (77.3)	
Intention to have more children	Yes	175 (88.4)	169 (85.4)	0.372
	No	23 (11.6)	29 (14.6)	
Companion utilization during childbirth	No	147 (74.2)	174 (87.9)	<0.001
	Yes	51 (25.8)	24 (12.1)	

The baseline levels of OV among postpartum women were found to be similar between the groups (intervention group 68.27 ± 13.16; control group 66.43 ± 11.27; *p* = 0.159); however, after the implementation of person-centered interventions, the level of OV in the intervention group decreased significantly more than that in the control group did (intervention 42.05 ± 6.97 vs. control group 66.45 ± 12.12; *p* < 0.000). According to these findings, person-centered interventions, including respectful maternity care workshops (RMCs), maternity open days (MODs), and maternal certificate of recognition (MCORs), were found to be effective in decreasing OV during facility-based childbirth. When the between-group score differences were analyzed, a statistically significant improvement in OV scores was observed in the experimental group after the implementation of the intervention (*p* < 0.001). In contrast, no significant difference was found in the control group (*p* = 0.924). The scores for OV decreased by an average of 26.00 points after the interventions were provided. This decrease was significantly greater than the average increase in the control group (0.02 points) (*p* < 0.001). Regarding elements of OV, a significant difference was observed between the intervention and control groups during the posttest (*p* < 0.001). In contrast, no statistical significance was found between the two groups during the pretest (Table 4).

**Table 4:** Effect of person-centered interventions on *o*bstetric violence (OV) in Southwest Ethiopia, 2024 (n1 = n2 = 198).

Variable	Group	Research period (mean score ± SD)		
		Pretest	Posttest	*p*-value^a^
Non-confidential care	Intervention	8.44 ± 3.14	5.40 ± 1.28	<0.001
	Control	8.62 ± 1.82	8.68 ± 2.41	0.632
	*p*-value^b^	0.494	< 0.001	
Undignified care	Intervention	21.78 ± 5.06	13.14 ± 1.87	<0.001
	Control	21.29 ± 3.84	21.23 ± 4.29	0.601
	*p*-value^b^	0.054	< 0.001	
Non-consented care	Intervention	12.88 ± 4.09	6.39 + 2.15	<0.001
	Control	12.04 ± 4.63	12.03 + 4.63	0.158
	*p*-value^b^	0.055	< 0.001	
Physical abuse	Intervention	11.86 ± 3.33	6.93 + 1.49	<0.001
	Control	11.72 ± 3.33	11.74 ± 3.22	0.687
	*p-*value^b^	0.671	< 0.001	
Discrimination	Intervention	9.86 ± 2.67	6.26 ± 1.80	<0.001
	Control	10.15 ± 2.55	10.17 ± 2.50	0.720
	*p-*value^b^	0.265	< 0.001	
Detention	Intervention	2.78 + 1.63	2.32 + 0.62	<0.001
	Control	2.61 + 1.57	2.60 + 1.55	0.319
	*p-*value^b^	0.287	0.017	
Overall obstetric violence sum mean scores	Intervention	68.05 ± 12.12	42.05 ± 6.97	<0.001
	Control	66.43 ± 11.27	66.45 ± 12.12	0.924
	*p-*value^b^	0.159	< 0.001	

The data are presented as the means (SD, standard deviation).

a Independent-samples *t*-test.

b Paired samples *t*-tests.

### Effects of the intervention on each type of obstetric violence

**Figure 2:**
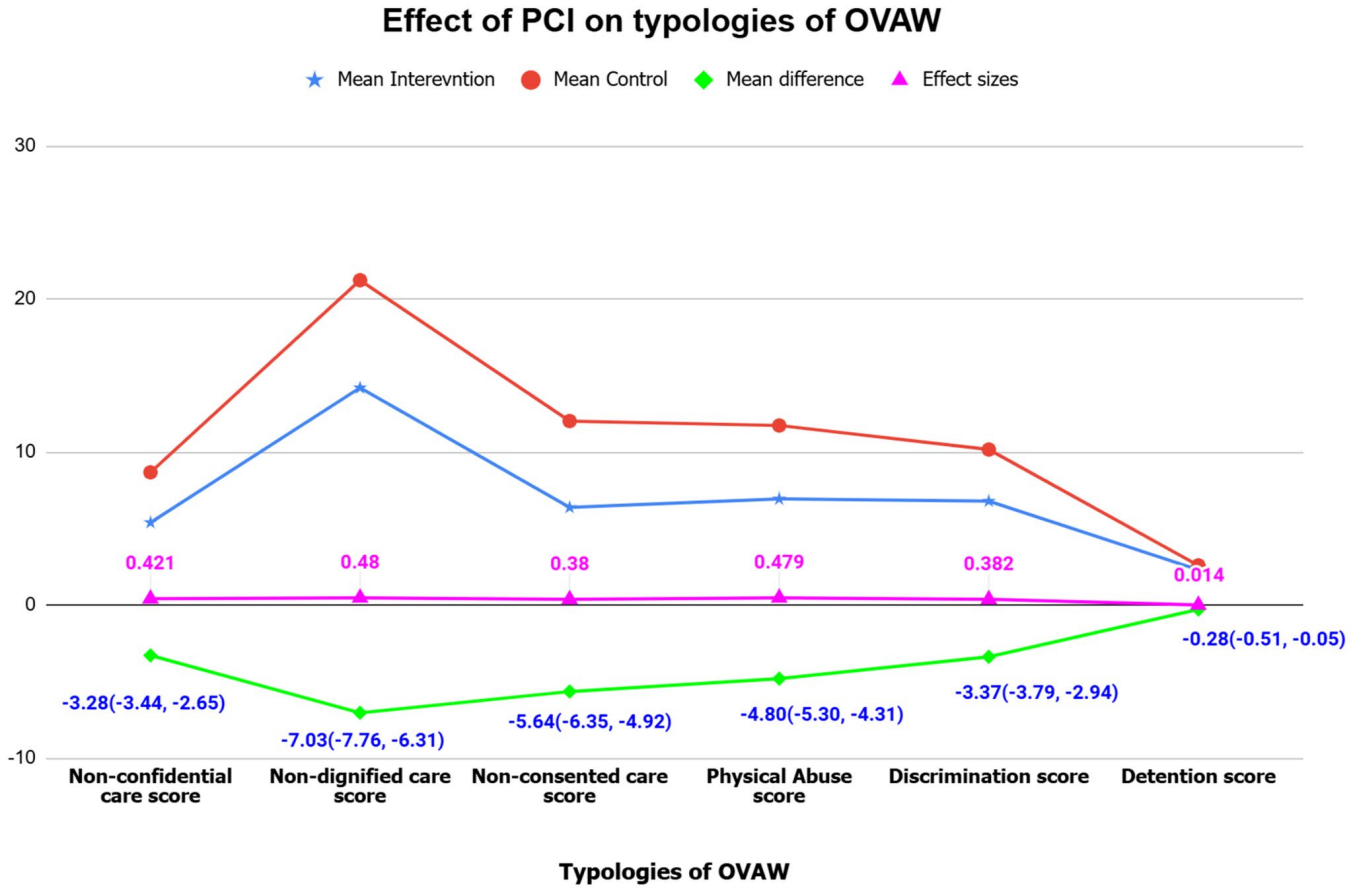
Multivariate general linear modeling parameters for the effects of person-centered interventions on obstetric violence (OV) typologies among postpartum women in *S*outhwest Ethiopia, 2024 (n1 = n2 = 198).

Figure 2 depicted the mean difference (MD) of non-confidential care (MD = −3.28; 95% CI −3.66, −2.90, effect size = 0.421), undignified care (MD = −7.03; 95% CI −7.76, −6.31, effect size = 0.48), non-consented care (MD = −5.64; 95% CI −6.35, −4.92, effect size = 0.38), physical abuse (MD = −4.80; 95% CI −5.30, −4.31, effect size = 0.479), discrimination (MD = −3.37; 95% CI −3.79, −2.94, effect size = 0.382), and detention (MD = −0.28; 95% CI −0.51, −0.05, effect size = 0.014). This finding indicated a significant decrease in OV typologies in the intervention group. Moreover, the highest impact of the intervention was observed for undignified care, with an effect size (ES) of 0.48, followed by physical abuse, 0.479. In contrast, the lowest impact was reflected in retention, with an effect size (ES) of 0.014.

The analysis of the bivariate general linear model revealed that the intervention and parity of the women predicted their degree of OV. Accordingly, for every 1-unit increase in the intervention group (i.e., being in the intervention group), the OV decreased by 23 points (*β* = −23.42; 95% CI: −25.40, −21.44). This means that women in the intervention group had OV scores that are, on average, 23 points lower than those in the control group. Another variable associated with the outcome variable (obstetric violence, OV) is antenatal care contact. The results indicated that being in the intervention group is linked to a decrease in OV by approximately seven points (*β* = −7.47; 95% CI: −4.055, 18.373). This suggested that women in the intervention group who had frequent contact with healthcare providers experienced, on average, seven fewer points of OV compared to those in the control group (Table 5).

**Table 5:** Univariate general linear modeling parameters for the independent factors of *o*bstetric violence (OV) among postpartum women in Southwest Ethiopia, 2024 (*N* = 396).

Variable	β	(95% CI)	*p*
Intervention status			
Control	Reference	Reference	ref
Intervention	−23.42	−25.40, −21.44	*p* < 0.001
Age	0.22	−0.17, 0.21	0.87
Education	−0.02	−0.18, 0.23	0.84
Parity			
Primipara	Reference	Reference	ref
Multipara	−1.17	−3.31, 0.97	0.29
Grand multipara	−2.46	−5.66, 0.74	0.13
Antenatal care contact			
1	Reference	Reference	ref
2–3	−1.04	2.179, 0.402	0.53
> = 4	−7.47	−4.055, 18.373	*p* < 0.001

CI, confidence interval.

## Discussion

Although there was no significant difference in the score of OV between the intervention and control groups at baseline in this study, a statistically significant difference in the score of OV between the intervention group and the control group at the end of the study. This decrease in OV indicates that person-centered intervention has a positive effect on changing this violence during facility-based childbirth. This study aimed to add to the existing strategies for reducing OV by examining the effects of person-centered interventions on OV during childbirth, thereby promoting a safer and positive childbirth experience and improving overall maternal health outcomes.

Although there were interventions to reduce OV or disrespect and abuse in Ethiopia, Kenya, and Tanzania, almost all of them underrepresented women in the intervention and lacked a comprehensive and multifaceted approach that integrates both healthcare providers’ and women’s perspectives (12, 13, 20, 21).

Furthermore, reducing OV during childbirth is a crucial global health initiative aimed at addressing mistreatment and abuse experienced by women. However, many intervention studies lack control groups, making it difficult to assess their effectiveness (12, 13, 17, 20, 21). To combat this issue, the WHO recommends comprehensive training for healthcare providers on respectful maternity care and empowering women through education about their rights (26). Implementing person-centered interventions such as respectful maternity care workshops, maternity open days, and maternal recognition certificates can help mitigate OV, improve safe and respectful care, and reduce barriers to healthcare services.

The current study revealed that 396 women who participated in this study experienced at least one form of OV. Studies conducted in Ethiopia showed that attitudes of healthcare providers, a lack of awareness of the universal rights of childbearing women, and cultural norms and expectations (normalizing disrespectful treatment) were significantly associated with OV or disrespect and abuse (1, 28–30). Another study among postpartum women in Ethiopia highlighted that educational status, ANC utilization, and complications during labor and childbirth were significantly associated with OV (4). Similarly, ANC contact and companion utilization were significantly correlated with OV in this study.

OV has a significant effect on birth outcomes, affecting both maternal and neonatal health (1, 2). The findings of this study show that participants experienced all forms of OV or disrespect and abuse at baseline. However, the mean scores for OV decreased significantly (*p* < 0.001) after the implementation of the intervention in the experimental group. In the control group, there was no significant difference (*p* > 0.05) in the mean score for OV between the pretest (66.43 ± 11.27) and posttest (66.45 ± 12.12). In contrast, in the intervention group, there was a significant difference (*p* < 0.001) in the mean OV score between the pretest (68.05 ± 12.12) and posttest (42.05 ± 6.97). The study also revealed that person-centered intervention had a significant effect (*p* < 0.001) on the mean scores of OV between the intervention group (42.05 ± 6.97) and the control group (66.45 ± 12.12) at the end of the study. The bivariate general linear model also revealed that, on average, women in the intervention group had OV scores that were 23 points lower than those of women in the control group. Similarly, a comparative before and after evaluation design conducted among women and healthcare providers at Injebara General Hospital, Northwest Ethiopia, revealed that the provision of training, staff motivation and other facility interventions significantly improved (*p* < 0.001) disrespect and abuse scores in the intervention group, from 71.8% at baseline to 15.9% at the endline, with a 55.9% change (17). Another study conducted in Kenya also indicated that provider training on respectful maternity care and linkages between the facility and community for accountability significantly reduced (*p* < 0.004) overall disrespect and abuse in the intervention group, from 20 to 13% (13). These notable changes in overall OV or disrespect and abuse reported in the three studies may be due to the combined effect of interventions that can address factors from healthcare providers’ and women’s perspectives.

In this study, the prevalence of various forms of OV, including undignified care, non-consented care, physical abuse, non-confidential care, discrimination, and detention, was significantly reduced at the endline compared to the baseline. Similar findings were observed in Southwest Ethiopia at Injebara Hospital, where interventions led to a decrease in the magnitude of disrespect and abuse elements such as physical abuse, non-consented care, non-confidential care, undignified care, discrimination, and neglected care. Additionally, a study in Kenya reported a 40–50% reduction in the typologies of disrespect and abuse among the intervention group (13, 17). These results highlight the effectiveness of targeted interventions in reducing OV across different settings.

The current scoping review of interventions aimed at addressing OV highlights multifaceted approaches that can be implemented together to reduce OV and promote positive childbirth experiences (2).

In recent years, training healthcare providers in respectful maternity care has gained attention globally (2, 26, 31). However, focusing solely on providers has not adequately addressed OV, as it often overlooks the involvement of vulnerable women. Despite Ethiopia’s efforts since 2016 to provide compassionate, respectful, and caring (CRC) training (18), significant improvements have not been observed (4), partly due to the lack of women-centered approaches and uniform training (12, 21). Our study addresses these gaps with a combined intervention that includes maternity open days and recognition certificates, as well as respectful maternity care workshops. This approach is feasible and recommended for low-income countries, like Ethiopia, to promote dignified care.

Evidences show that workshop programs emphasizing respectful maternity care (RMC) effectively address the root causes of disrespect and abuse in maternity settings. These programs clarify values and transform the attitude of healthcare providers, help the healthcare providers and the women to exercise their rights and responsibilities during childbirth, enhance communication skills, foster an environment of empathy and respect toward birthing women, and improve the quality of maternal healthcare (14, 17). The current study supports this by showing that structured training leads to a significant decrease in OV during delivery services, improving women’s care experiences.

The current study revealed that the maternity open days event engages the women and educates them about their rights and responsibilities, care standards, and the nature of OV. It demystifies childbirth, reduces anxiety, and fosters a positive experience by familiarizing women with facilities and staff. Moreover, promote mutual respect between women and healthcare providers, leading to a decrease in the experience of OV, and encourage facility-based deliveries, thereby improving maternal care quality at service delivery points. This is supported by the study conducted in Tanzania (20).

In addition, the systematic review underscores that disrespectful and abusive behaviors in maternity care settings are often rooted in systemic issues, including inadequate provider training, understaffing, healthcare providers’ negative behaviors/attitudes, cultural factors, and a lack of autonomy or accountability (2, 32). Therefore, interventions such as RMC training and maternity open days are critical not only for immediate improvements in care but also for fostering a culture of respect and accountability within healthcare systems.

This study reveals that issuing maternal certificates of recognition to postnatal women can significantly reduce OV and disrespect during childbirth. This practice acknowledges and empowers women, fostering better interactions with healthcare providers and a more respectful care environment. It validates their experiences, encouraging them to report mistreatment and mitigates negative mental health outcomes such as postpartum depression, by promoting a supportive environment, as stated by other studies (33, 34). This aligns with studies showing that respectful communication can reduce mistreatments and postpartum PTSD (35). Respectful communication involves several key components, such as fully engaging with the woman’s concerns, questions, and preferences, understanding of the woman’s emotional and physical experience during pregnancy, labor, and postpartum care. Using kind, supportive language to acknowledge fears, pain, or vulnerabilities, providing accurate, jargon-free explanations about procedures, Acknowledging the woman’s right to make decisions about her care, recognizing and respecting the woman’s cultural, religious, or personal values and tailoring communication to align with her preferences, using neutral, supportive language and involving the woman’s partner, family, or support persons. These elements ensure that interactions between healthcare professionals, women, and their families are empathetic, empowering, and conducive to trust and dignity. Therefore, integrating maternal certificates of recognition with existing recognition programs can promote respectful maternity care.

The qualitative insights further illustrate that patient-centered care, which includes respectful and empathetic interactions, is essential in combating disrespect and abuse during childbirth (36). This is echoed by the World Health Organization, which identifies respectful maternity care as a key strategy for improving maternal health services globally (37). The emphasis on respectful interactions not only enhances the immediate childbirth experience but also has long-term implications for women’s willingness to seek care in the future, as evidenced by the findings of (38).

Person-centered interventions (respectful maternity care training, maternity open days, and certificates of recognition) can effectively contribute to the reduction of OV and instances of disrespect and abuse during childbirth. These strategies empower women, enhance provider attitudes, and promote a culture of respect within maternity services, ultimately leading to improved maternal health outcomes.

In our study, the chi-square test results indicate that the use of companions (a woman’s family, spouse/partner, or a female friend) during childbirth is more prevalent in the intervention group compared to the control group. This suggests that women who have a companion present during facility-based childbirth are less likely to experience OV than those without a companion. A study conducted in Ethiopia supports this finding, indicating that the presence of a companion (such as a woman’s family, spouse/partner, or a female friend) can enhance respectful maternity care (39). Companions (such as a woman’s family, spouse/partner, or a female friend) offer numerous benefits, including emotional, informational, and physical support, which lead to improved maternal and neonatal outcomes. They help reduce anxiety, postpartum depression, and OV, while enhancing mother–baby bonding and breastfeeding. Additionally, companions act as advocates, empowering women to feel more in control during labor, thereby improving the quality of care and overall experience (40, 41). Despite these advantages, the utilization of companions remains low, underscoring the need for clear guidelines to support and promote companionship in healthcare settings. This utilization of companions (woman’s family, spouse/partner, or a female friend) during childbirth is a critical intervention to reduce OV, which fosters respectful maternity care by improving communication, increasing accountability, enhancing emotional support, and protecting women’s rights and dignity. Therefore, health systems should be encouraged to facilitate companion presence as part of comprehensive strategies to combat OV and improve positive childbirth experiences.

The bivariate general linear model shows that intervention status and ANC contact (≥4) were significantly associated with OV during facility-based childbirth. Empirical research highlights that OV has a negative effect on the utilization of ANC (1, 3, 4). However, in this study, due to the implementation of robust and comprehensive interventions, women who had four or more ANC contacts were less likely to experience OV than women who had fewer than four ANC contacts. This finding is in agreement with a study conducted in Ethiopia, which reported a decrease in disrespect and abuse by half a percent (17). The possible justification for the reduction in both studies would be the positive effect of the selected interventions in mitigating the OV or disrespect and abuse that the women had experienced, and the provision of respectful and dignified maternal healthcare services to the women.

## Conclusion

Person-centered interventions, which constitute respectful maternity care, maternity open days, and maternal certificates of recognition, were effective in reducing OV and its typologies during childbirth in the study area. Almost all women who underwent the intervention, on average, received respectful and dignified care over the intrapartum period because of the reduction in violence. This intervention strategy could also help mitigate the magnitude of OV during antenatal care and improve the utilization of companions during facility-based childbirth.

### Recommendations

Stakeholders such as government institutions, non-governmental organizations and international and scientific communities who are working on maternal and child health issues should ensure the active participation of women in all programs/interventions so that the incidence of OV can be substantially minimized, respectful maternal care can be promoted, and maternal and child health outcomes can be enhanced. The government of Ethiopia should integrate person-centered interventions, particularly maternity open days and maternal certificates of recognition, into existing maternal health strategies to empower women, improve women’s health-seeking behavior, and create a positive caring environment. It is also crucial to scale up intervention strategies in maternal healthcare, such as antenatal care, and other healthcare facilities.

### Strengths and limitations

This is the first interventional study explicitly conducted on OV in Africa, including Ethiopia. In the best search of the literature, with the exception of two studies from Africa, none of them developed strategies that accommodate and involve women in the intervention process or considered a control group to evaluate the effect of the intervention (16, 42). However, the current study included women and a control group during the research process. One of the drawbacks of this study could be the use of different study populations for baseline and endpoint surveys despite their similar background characteristics. This may cause internal validity problems. The structural part of the health system and community-related issues, focusing on sociocultural factors, were not addressed in this study.

## Data Availability

The raw data supporting the conclusions of this article will be made available by the authors, without undue reservation.
